# Effect of Direct Alkyne Substitution on the Photophysical Properties of Two Novel Octasubstituted Zinc Phthalocyanines

**DOI:** 10.1002/open.202300295

**Published:** 2024-01-26

**Authors:** Hande Pekbelgin Karaoğlu

**Affiliations:** ^1^ Department of Chemistry Faculty of Science and Letters Istanbul Technical University Maslak 34469 Istanbul Turkey

**Keywords:** phthalocyanine, alkynyl-substituted, photophysical properties, octa substitute, zinc

## Abstract

The synthesis of two novel phthalonitrile derivatives (**3**–**4**) bearing ethynylcyclohex‐1‐ene and ethynylcyclohexane groups and two peripherally octa substituted zinc (II) phthalocyanines (**5**–**6**) were prepared. The synthesis of phthalonitrile derivatives was performed with Sonagashira coupling reaction by using palladium‐catalyzed. The newly synthesized compounds were characterized by using FT‐IR, NMR, mass, and UV‐Vis absorption spectroscopy techniques. Aggregation studies of **5** and **6** were performed in various organic solvents and different concentrations in tetrahydrofuran (THF). The photophysical studies of the Pcs were performed in THF to determine the effect of the alkyne groups on the fluorescence of the Pc ring. Substances showing fluorescence properties can be used in practical applications such as to create an image in microscopy. Fluorescence quantum yield (Φ_F_) and fluorescence lifetime (τ_F_) of **5**–**6** were calculated. The fluorescence quenching studies of **5**–**6** were performed by adding the different concentrations of 1,4‐benzoquinone (BQ) to a constant concentration of the Pcs in THF and it was found that benzoquinone was an effective quencher. The values of the Stern‐Volmer constant (K_sv_) and quenching constant (k_q_) of zinc phthalocyanines (**5**–**6**) were examined. All obtained results were compared with each other and with unsubstituted zinc Pc compound used as a reference.

## Graphical abstract

The synthesis and characterization of two new alkynyl‐substituted phthalonitrile derivatives and their peripherally octasubstituted zinc phthalocyanine (ZnPc) compounds were reported. The aggregation behavior of phthalocyanines was investigated. Fluorescence quantum yields, lifetimes, and quench studies of phthalocyanines were examined. The photophysical parameters obtained for synthesized phthalocyanines were compared with each other and with the unsubstituted ZnPc used as a standard.

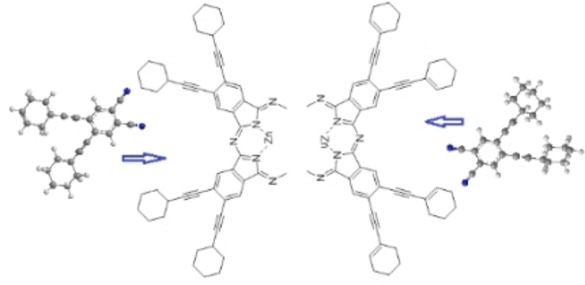

## Introduction

Phthalocyanines (Pcs) and porphyrins have several photophysical, photochemical, and photo‐biological properties making them effective aromatic macrocyclic compounds for different purposes.[^1^, ^2^] Pcs, which can be synthesized simply as a result of cyclotetramerization reactions of phthalonitrile compounds, can be modified to change emission wavelengths, hydrophilicity, and absorption for use in different applications.[^2^, ^3^] Numerous information about their synthesis, photophysical, and photochemical properties have been revealed since their discovery in 1907.[^1^, ^2^, ^4^] They are now industrially prepared and used as dyes and colorants and a lot of patents and reports have been published for their application as liquid crystals,^[5]^ boron neutron capture therapy,[^6^, ^7^] electrochromic compounds,^[8]^ nonlinear optical materials,^[9]^ photovoltaic cells,^[10]^ chemical sensors^[11]^ and catalysts in organic synthesis.^[12]^ The photophysical properties of Pcs are of great interest because they exhibit very strong absorption peaks at longer wavelengths in the far‐red region of the visible spectrum than porphyrins. Their photophysical properties can be fine‐tuned through the addition of substituents to the periphery or nonperiphery of the macrocycle, or by changing the nature of the metal in the center.[^13^, ^14^] Based on all these properties, Pcs can be shown potential for photodynamic cancer therapy (PDT).[^15^, ^16^, ^17^] However, since most of the Pcs have poor solubility in various organic solvents, including chloroform (CHCl_3_), dichloromethane (DCM), toluene, ethyl acetate (EtAc), ethanol (EtOH), methanol (MeOH), THF, and especially in water, their purification and application are limited. The solubility of the phthalocyanines can be increased by the binding of long alkyne chains, bulky substituents, or water‐soluble groups to the macrocycle's peripheral or non‐peripheral positions or by the use of isomeric mixtures.[^7^, ^18^, ^19^, ^20^, ^21^, ^22^] While tetrasubstituted Pcs are obtained as a mixture of four different isomers that differ in asymmetry (D_2h_, C_4h_, C_2v_ and C_s_), octasubstituted derivatives are synthesized isomerically pure. The use of isomeric mixtures is not preferred because it causes difficulties in the reproducibility of photochemical, photophysical, and biological measurements. By using 4,5 or 3,6‐disubstituted starting materials, a single isomer can be obtained because mono‐substituted phthalonitrile derivatives cause a mixture of isomers.[^6^, ^7^, ^9^, ^10^, ^14^, ^22^, ^23^]

The functionality of the Pc can be altered by the formation of a C−C bond that binds directly to the Pc ring by cross‐coupling reactions. Although there are studies in the literature on Pc carrying a terminal alkyne group, studies on the synthesis of compounds bearing the alkyne group directly linked to the Pc ring are extremely limited. According to the literature, the synthesis of octa alkynyl phthalocyanines was accomplished by Leznoff and coworkers starting from halophthalonitriles.[^24^, ^25^] A review of alkynyl‐substituted phthalocyanines was published in 2006 by Torres et al.^[26]^ The number of octasubstituted Pcs reported in this review is very limited. Of these, only three studies belong to octasubtitue Pc compounds synthesized from halophthalonitriles, reported by Leznoff et al.[^24^, ^25^] and Dennis K. P. Ng et al.^[27]^ Photophysical measurements of octasubstituted alkynyl Pcs containing alkyl groups of various lengths synthesized in these studies were not performed. In a study reported by Bayır et al. in 2015, the fluorescent properties of tetra‐alkynyl Pc compounds were compared.^[28]^ Another study published by Bayır et al. is about fluorescence measurements of an alkynyl‐substituted asymmetric Pcs.^[29]^ In another study performed by the same group, alkynyl‐substituted phthalocyanines were designed and their biological activities were examined.[^30^, ^31^] In addition, up to now, there are no studies in the literature on the fluorescence efficiency of octa‐alkynyl‐substituted Pcs.

Fluorescence occurs during the transition of an electron or molecule from the excited state to the ground state. The specific frequencies of excitation and emission vary depending on the molecule or atom. Generally, the emitted light has a lower photon energy (longer wavelength) than the absorbed light. The lifetime of the electron in the excited state is short and is usually on the order of 10^−8^ seconds. When the radiation source stops, the fluorescent molecules stop glowing almost immediately. Therefore, the fluorescence lifetime is an important parameter for practical applications of fluorescence. Phthalocyanine derivatives have a conjugated 18 π electron system and can exhibit different fluorescence properties depending on the type of metal and substituents.[^3^, ^13^, ^16^, ^17^, ^23^, ^27^, ^28^, ^29^] Substances that exhibit fluorescent properties have the potential to create an image in laser scanning confocal microscopy.

In this study, the synthesis and characterization of two new phthalonitrile derivatives bearing ethynylcyclohex‐1‐ene and ethynylcyclohexane groups and their peripherally octasubstituted zinc phthalocyanine (ZnPc) compounds were reported. The new phthalonitrile derivatives (**3**–**4**) were synthesized from the reaction of diiodophthalonitrile with 1‐ethynylcyclohex‐1‐ene and ethynylcyclohexane respectively in the Sonagashira coupling reaction. Palladium‐catalyzed cross‐coupling reactions are important for the functionalization of phthalocyanines by binding to different groups such as alkynyl and alkenyl. The aggregation behavior of compounds **5** and **6** was investigated by absorption measurements at different concentrations in THF and the same concentrations of various organic solvents EtAc, dimethylformamide (DMF), dimethylsulfoxide (DMSO), THF, and toluene. To the best of our knowledge, there is no study reported on the investigation of the fluorescence efficiency and photophysical properties of octasubstituted Pc starting from halophthalonitrile in which the Pc ring directly contains an alkyne group. Fluorescence quantum yields, lifetimes, and quench studies of ZnPcs (**5**, **6**) were examined in THF. The photophysical parameters obtained for the newly synthesized ZnPcs were compared with each other and with the unsubstituted ZnPc used as a standard. Additionally, the fluorescence quantum yields of compounds **5** and **6** were compared to other Pcs substituted alkyl groups in the literature.[^28^, ^29^] The photophysical measurements of the substituted ZnPcs were performed to determine the effect of the ethynylcyclohex‐1‐ene and ethynylcyclohexane groups on the fluorescence of the Pc ring. Therefore, it was decided to study the photophysical properties of new octasubstituted ZnPcs containing direct alkyne groups.

## Materials and Methods

### Materials and apparatus

1‐ethynylcyclohex‐1‐ene, cyclohexylacetylene, Pd(PPh_3_)_2_Cl_2_, CuI and Zn(CH_3_COO)_2_ were obtained from Sigma‐Aldrich, Germany. 4,5‐diiodophthalonitrile was synthesized starting from o‐xylene as given in the literature.[^32^, ^33^, ^34^] All solvents used during the synthesis and purification of the final products were obtained from Sigma‐Aldrich.

A Perkin‐Elmer Spectrum One spectrometer was used to obtain FT‐IR spectra of the synthesized compounds. NMR spectra of the compounds were recorded using an Agilent V NMRS 500 MHz spectrometer. Bruker Microflex LT MALDI‐TOF MS spectrometer was used to obtain mass spectra of synthesized compounds. UV spectra of Pc compounds were obtained using a Scinco LabProPlus UV/Vis spectrophotometer. A Perkin Elmer LS55 fluorescence spectrophotometer was used for fluorescence measurements of Pc compounds. It was determined that the compounds obtained as a result of spectroscopic measurements used confirmed the designed compounds.

### Synthesis

#### 4,5‐bis(cyclohex‐1‐en‐1‐ylethynyl)phthalonitrile (3)

500 mg 4,5‐diiodophthalonitrile (2) (1.32 mmol)[^32^, ^33^, ^34^] and 335.30 mg 1‐ethynylcyclohex‐1‐ene (3.16 mmol) were dissolved in 150 ml of dry Et_3_N: toluene (1 : 1) mixture. The reaction mixture was effectively deoxygenated using nitrogen. Catalytic amounts of Pd(PPh_3_)_2_Cl_2_ (46.2 mg, 65.8 μmol) and CuI (16.7 mg, 87.7 μmol) were added to the reaction mixture. After the reaction content was stirred and heated at 80 **°C** for 16 h, the solvent was evaporated. The solid product obtained was chromatographed on silica gel with 1 : 1 dichloromethane (DCM): hexane as a mobile phase. Then the last purification was accomplished by crystallization with MeOH. Yield: 285 mg (64.4 %); FT‐IR ν (cm^−1^): 3023 (CH, aromatic), 2915 (CH, aliphatic), 2233 (C≡N), 2191 (C≡C) 1620 (C=C); ^1^H NMR (500 MHz, CDCl_3_): ppm, 7.75 (2H, s), 6.38 (2H, m), 2.26–2.18 (8H, m), 1.74–1.63 (8H, m); ^13^C NMR (125 MHz CDCl_3_): ppm, 139.2, 135.9, 131.1, 120.2, 114.9, 113.3, 102.4, 83.5, 28.7, 26.0, 22.1, 21.3.

#### 4,5‐bis(cyclohexylethynyl)phthalonitrile (4)

500 mg 4,5‐diiodophthalonitrile (2) (1.32 mmol)[^32^, ^33^, ^34^] and 341.7 mg cyclohexylacetylene (3.16 mmol) were dissolved with dry Et_3_N: toluene (1 : 1) mixture and filled into a 250 mL pressure tube. The solution mixture was bubbled with nitrogen for about 30 minutes. After adding a catalytic amount of Pd(PPh_3_)_2_Cl_2_ (46.2 mg, 65.8 μmol) and CuI (16.7 mg, 87.7 μmol), the mixture was stirred at 80 ° C for 16 hours. After the reaction was terminated, the solvent was evaporated. The purification of crude product was accomplished by column chromatography on silica gel using a 3: 1 hexane: DCM mixture followed by crystallization with MeOH. Yield: 90 mg (20.1 %); FT‐IR ν (cm^−1^): 3035 (CH, aromatic), 2934 (CH, aliphatic), 2235 (C≡N), 2209 (C≡C), 1592 (C=C); ^1^H NMR (500 MHz, CDCl_3_): ppm, 7.75 (2H, s), 2.73–2.69 (2H, m), 1.89–1.87 (4H, m), 1.80–1.77 (4H, m), 1.61–1.56 (6H, m), 1.44–1.39 (6H, m); ^13^C NMR (125 MHz, CDCl_3_): ppm, 136.4, 131.7, 114.9, 113.2, 106.0, 77.6, 32.1, 29.9, 25.8, 24.6.

#### 2,3,9,10,16,17,23,24‐octakis‐4,5‐bis(cyclohex‐1‐en‐1‐ylethynyl)phthalocyaninato zinc(II) (5)

Under nitrogen atmosphere, 150 mg 4,5‐bis(cyclohex‐1‐en‐1‐ylethynyl)phthalonitrile (3) (0.45 mmol) and 20.5 mg Zn(CH_3_COO)_2_ (0.11 mmol) were stirred and dissolved in 5 mL of N,N‐dimethylaminoethanol (DMAE). The reaction mixture was heated to 135 **°C** and the reaction was continued at this temperature for 24 h. After cooling to room temperature, the reaction mixture was poured into ice‐cold water, precipitated, filtered, and washed several times with hot MeOH. The greenish product was obtained by chromatography method on silica gel using DCM: MeOH (50 : 1 V/V). Yield: 98 mg (62.3 %); Anal. calcd for C_96_H_80_N_8_Zn: C, 81.71; H, 5.71; N, 7.94; Found: C, 81.73; H, 5.68; N, 7.90 %; FT‐IR ν (cm^−1^): 3107 (CH, aromatic), 2928 (CH, aliphatic), 2191 (C≡C), 1601 (C=C), (C) ; UV‐Vis (THF): λ_max_, nm (log ϵ): 384 (4.67), 721 (5.27). MS (MALDI‐TOF) m/z: 1408.5 [M]^+^; ^1^H NMR (500 MHz, CDCl_3_): ppm, 8.89 (8H, s), 6.54 (8H, b), 2.56 (16H, b), 2.35 (16H, b), 1.87 (16H, b), 1.79 (16H, b).

#### 2,3,9,10,16,17,23,24‐octakis‐4,5‐bis(cyclohexylethynyl)phthalocyaninato zinc(II) (6)

4,5‐bis(cyclohexylethynyl)phthalonitrile (4) (100 mg, 0.29 mmol) and 13.47 mg Zn(CH_3_COO)_2_ (0.073 mmol) were mixed thoroughly in 5 mL of DMAE at 135 °C under nitrogen atmosphere. The reaction was terminated after 24 hours, after the mixture was cooled to room temperature, it was settled by pouring into cold water and filtered. The crude product was washed several times with hot MeOH and hexane until the filtrate was colorless. Purification was completed by column chromatography over silica gel (50: 1 DCM: MeOH was used as eluent). Yield: 48 mg (45.8 %); C_96_H_96_N_8_Zn: C, 80.79; H, 6.78; N, 7.85; Found: C, 80.82; H, 6,81; N, 7.82 %; FT‐IR ν (cm^−1^): 3006 (CH, aromatic), 2989 (CH, aliphatic), 2204 (C**≡**C),1596 (C=C), (C); UV‐Vis (THF): λ_max_, nm (log ϵ): 352 (4.32), 678 (4.74). MS (MALDI‐TOF) m/z: 1425.7 [M+H]^+^; ^1^H NMR (500 MHz, CDCl_3_): ppm, 9.13 (8H, s), 2,95 (8H, m), 2.12–2.11 (16H, m), 2.00 (16H, b), 1.85–1.84 (16H, m), 1.54 (32H, b).

### Photophysical parameters

Fluorescence quantum yields (Φ_F_) of the ZnPc derivatives in THF were calculated using the comparative method given in Eq. (1) as in the literature.[^23^, ^35^] In this comparative method, the quantum yields of the newly synthesized Pc compounds were determined by using unsubstituted ZnPc as the standard compound (Φ_F_=0.17 in DMF).[^23^, ^35^] The area under the fluorescence emission curves for the newly synthesized ZnPcs (**5**, **6**) and the standard is expressed in equation 1 as F and F_std_. A and A_std_ represent the absorbances of ZnPc numbered **5**, **6**, and ZnPc used as standard. n and n_std_ are the refractive indices of the solvent (THF) used for compounds **5**, and **6** and the solvent (DMF) used for the standard ZnPc compound, respectively.

(1) $$\emptyset_{F}=\emptyset_{F}\left(Std\right)\frac{FA_{Std}n^{2}}{F_{Std}An_{Std}^{2}}\;$$

PhotochemCAD program which uses the Strickler‐Berg equation was used to determine the natural radiative lifetime (τ_0_) and the fluorescence lifetime (τ_F_) (Eq. 2.[^23^, ^36^]

(2) $$\emptyset_{F}=\frac{\tau_{F}}{\tau_{0}}\;$$

Fluorescence quenching studies were accomplished in THF by adding varying concentrations of BQ (0, 0.008, 0.016, 0.024, 0.032, and 0.040 mol dm^−3^) to solutions of ZnPc compounds at constant concentration (4.00 μmol dm^−3^). In the presence of BQ, an energy transfer occurred between the BQ and ZnPc molecule (fluorophore), and all spectra were recorded after BQ addition.

K_SV_ is the Stern‐Volmer constant was calculated using Stern‐Volmer (SV) equation Eq. 3. In this equation, I_o_ is the fluorescence intensities of ZnPc and I is the fluorescence intensities of ZnPc after adding different concentrations of BQ.

K_SV_ is determined from the slope of the graph of the I_o_/I ratios against [BQ] according to Eq. 3.

(3) $$\frac{I_{0}}{I}=1+\;K_{SV}\left[BQ\right]\;$$

The bimolecular quenching constant (kq) was calculated by quenching ZnPcs with BQ in by using Eq 4.

(4) $$K_{SV}=k_{q}\times \tau_{F}\;$$

## Results and Discussion

**Scheme 1:**
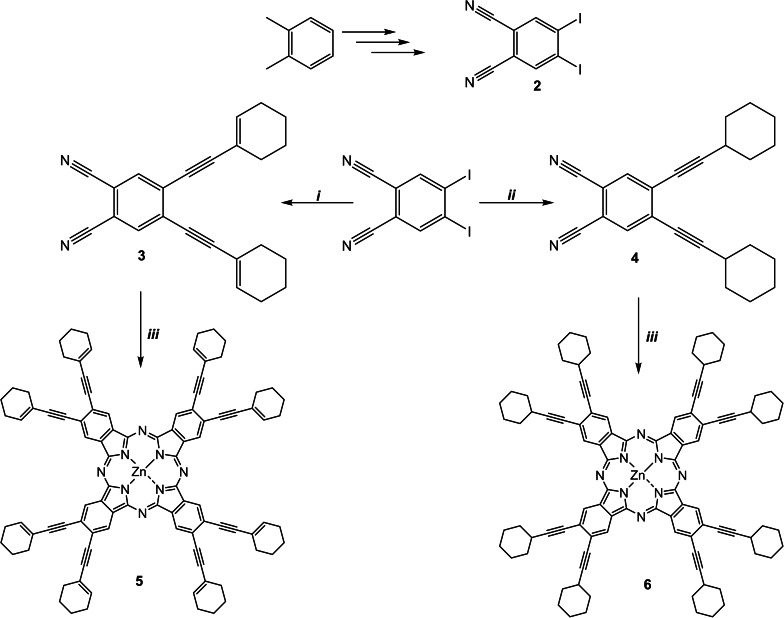
The synthetic route of compounds (**2**–**6**). (i) 1‐ethynylcyclohex‐1‐ene, Pd(PPh_3_)_2_Cl_2_, CuI, Et_3_N:toluene, 80 °C, 16 h; (ii) cyclohexylacetylene, Pd(PPh_3_)_2_Cl_2_, CuI, Et_3_N:toluene, 80 °C, 16 h; (iii) DMAE, Zn (CH_3_COO)_2_, 135 °C, 24 h.

The steps involved in the synthesis of novel phthalonitrile derivatives (**3**, **4**) and their corresponding Pc derivatives (**5**, **6**) were depicted in Scheme 1. 4,5‐diiodophthalonitrile (**2**) was synthesized according to the literature.[^32^, ^33^, ^34^] Compounds **3** and **4** were synthesized as a result of the Sonogashira coupling reaction between diiodo phthalonitrile and 1‐ethynylcyclohex‐1‐ene (for **3**) and cyclohexylacetylene (for **4**) at 80 °C by using Pd catalyst. The synthesis of compounds **5** and **6** was carried out under an N_2_ atmosphere at 135 °C in DMAE by cyclotetramerization of compounds **3** and **4**. Compounds **3** and **4** were purified by column chromatography over silica gel using DCM: hexane as eluent, followed by crystallization with MeOH. Compounds **5** and **6** were purified by applying a column chromatography method over silica gel (using 50: 1 DCM: MeOH as eluent). The novel compounds were characterized using diverse methods including FT‐IR, UV‐Vis, NMR, and Mass spectroscopic techniques. The results obtained confirmed all of the proposed structures.

**Figure 1:**
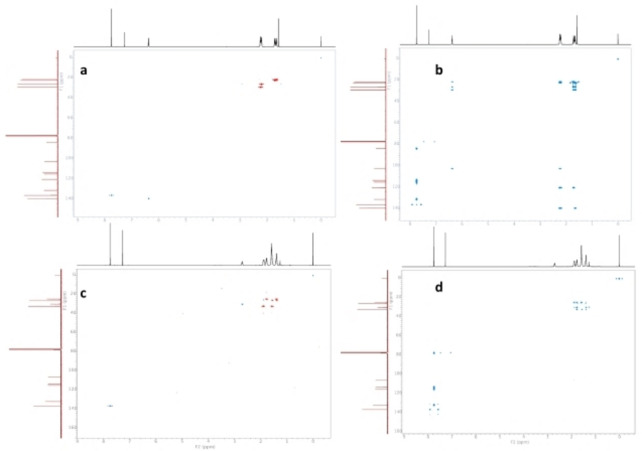
^1^H‐^13^C HSQC (a for **3**, c for **4**) and ^1^H‐^13^C HMBC (b for **3**, d for **4**).

The main difference between the FT‐IR spectra of phtalonitrile compounds and Pcs was the peak of the nitrile group observed in compounds **3**, and **4** at 2233 cm^−1^ and 2235 cm^−1^ respectively. After conversion of compounds **3** and **4** to compounds **5**, and **6** by the cyclotetramization reaction, the peak at about 2233 cm^−1^ of the functional group C≡N in phthalonitrile compounds disappeared. This result was the first proof that compounds **5**, and **6** were formed. In the IR spectra, aromatic C−H groups were observed at 3023 cm^−1^ for **3**, 3035 cm^−1^ for **4**, 3107 cm^−1^ for **5**, and 3006 cm^−1^ for **6**. Compounds **3**–**6** showed the aliphatic C−H protons at 2915 cm^−1^, 2934 cm^−1^, 2928 cm^−1^ and 2989 cm^−1^ respectively. The stretching peaks of C≡C groups of compounds **3**–**6** were observed at about 2190 cm^−1^. Also, the peaks of C=C functional groups were detected similar to each other at about 1600 cm^−1^ for **3**–**6**. The peaks observed in the FT‐IR spectra of the synthesized materials confirm the proposed structures. ^1^H‐NMR spectra of compound **3** and its corresponding compound **5** were similar as expected. The protons of the aromatic ring appear at 7.75 ppm for compound **3** and 8.89 ppm for compound **5** as singlets. Peaks of olefinic hydrogens in the cyclohexene ring were observed for **3** as multiplet signals at 6.38 ppm and for **5** as a broad peak at 6.54 ppm. In the ^1^H‐NMR spectrum of **3**, aliphatic protons were observed as multiples at 2.26–2.18 ppm and as multiples at 1.74–1.63 ppm. Similarly, protons belonging to the same group in compound **5** were observed at 2.56 ppm, 2.35 ppm, 1.87 ppm, and 1.79 ppm as broad peaks. ^1^H‐^13^C HSQC (Figure 1 (a)) and ^1^H‐^13^C HMBC (Figure 1 (b)) spectra of compound **3** showed well‐resolved correlations and ^13^C chemical shift values assigned evaluating these spectra.

^1^H‐NMR spectra of **4** and **6** were similar as expected also. Aromatic protons were observed at 7.75 ppm for compound **4** and 9.13 ppm for compound **6** as singlets. The peaks of the aliphatic H of the cyclohexyl ring in compound **4** were observed at 2.73–2.69 ppm, 1.89–1.87 ppm, 1.80‐1.77 ppm, 1.61–1.56 ppm, and 1.44–1.39 ppm, as multiplet peaks. The aliphatic protons of the cyclohexyl group in compound **6** were detected as multiplets at 2.95 ppm, 2.12–2.11 ppm, 2.00 ppm, 1.86–1.84 ppm, and 1.54 ppm respectively, in accordance with the structure. ^1^H‐^13^C HSQC (Figure 1 (c)) and ^1^H‐^13^C HMBC (Figure 1 (d)) spectra of compound **3** showed well‐resolved correlations and ^13^C chemical shift values assigned evaluating these spectra.

Characteristic UV‐Vis spectra of Pcs include the Q band (at around 600–700 nm) and the B band (also referred to as the Soret band, at around 300–400 nm) resulting from π–π* transitions. The UV‐Vis spectrums of **5** and **6** were recorded in THF. In their spectrums, the B bands were observed at 384 nm for **5**, 352 nm for **6**, while the Q bands were observed at 721 nm for **5**, 678 nm for **6**.

The molecular ion peaks of **5** and **6** appear at m/z=1408.5 [M]^+^ and 1425.7 [M+H]^+^, respectively and the results confirm the characterization of the synthesized compounds.

### Aggregation studies

**Figure 2:**
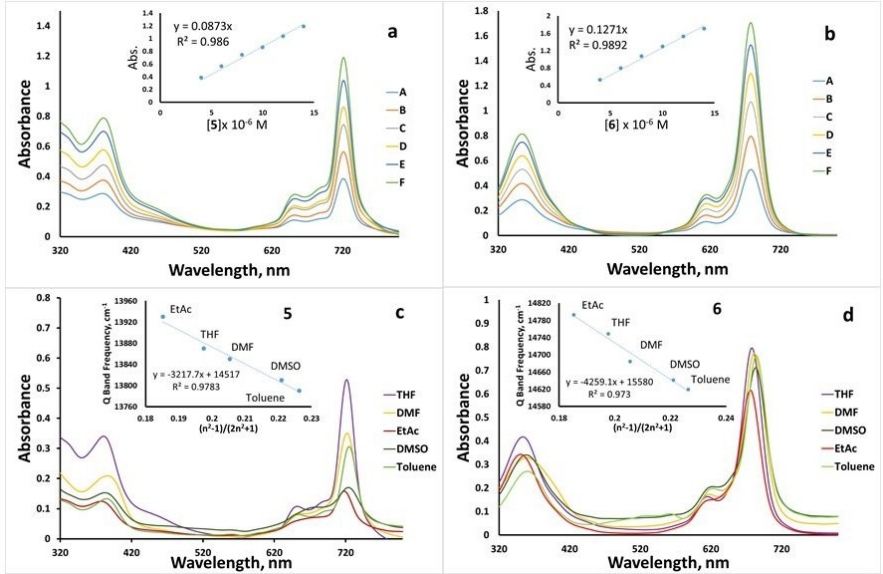
Aggregation behavior of novel ZnPcs **5** (a) and **6** (b) at different concentrations from 4×10^−6^−14×10^−6^ M in THF and plot of the absorbance against the concentration of **5** (insert figure a) and **6** (insert Figure b). UV‐Vis spectra of **5** (c) and **6** (d) in various solvents (concentration; 6×10 ^−6^) and plot of the Q band frequency against (n^2^−1)/(2n^2^+1) of **5** (insert Figure c) and **6** (insert Figure d).

The aggregation of Pcs is known as the stacking of rings starting from the monomer towards the dimer and higher‐order complexes Aggregation behavior is generally dependent on solvent, substituents, central metal ions, concentration, and temperature.[^1^, ^37^, ^38^, ^39^] Aggregation in phthalocyanine complexes is undesirable because aggregates are typically photoinactive. Within the scope of this study, an aggregation study using THF as a solvent for compounds **5** and **6** in different concentration ranges (4×10^−6^−14×10^−6^ M) was carried out (Figure 2a, 2b). In the investigated concentration range, it was determined that the absorption increased in parallel with the concentration of the solution. According to the Lambert‐Beer law, the slopes of the absorption versus concentration graphs were linear and no new peaks were observed as the concentration increases, indicating that aggregation tendency was not detected. The absorption spectra of compounds **5** and **6** in various organic solvents were also investigated (Figure 2c, 2d). It was observed that the Q band was redshifted depending on the refractive index of the preferred solvent. The bathochromic shift of the Q band for **5** and **6** in the UV spectrum was observed in the order EtAc<THF<DMF<DMSO<toluene depending on the numerical value of the refractive index for **5** and **6** (Figure 2c, 2d). The Bayliss technique was used to investigate the electronic absorptions of **5** and **6** in these organic solvents.^[40]^ Insert Figure 2c, 2d to show the frequency of the Q band plotted against the (n^2^−1)/(2n^2^+1) function. The linearity of the graph in insert Figure 2c, 2d confirms that the redshifts in the Q band frequency are mostly due to the solvation effect.

### Fluorescence quantum yields and lifetimes

**Figure 3:**
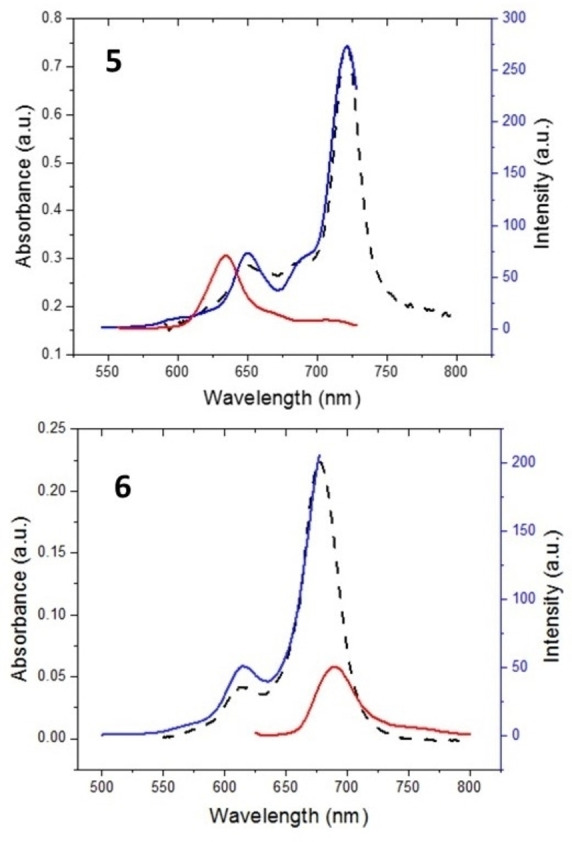
Absorption (…), excitation (blue), and emission (red) spectra of **5** and **6** in THF (4×10^−6^ M).

The fluorescence behavior of the synthesized ZnPcs was investigated in THF. The fluorescence emission, consequent consequent excitation, and absorption spectra were given in Figure 3a (for **5**) and 3b (for **6**). The Q bands, characteristic for Pcs, were observed at 721 nm (log ϵ was 5.27) for **5** and at 678 nm (log ϵ was 4.74) for **6**. The emission peak of **5** was detected at 734 nm as a result of the excitation at 647 nm wavelength. The emission peak for **6** was observed at 690 nm, upon excitation at 615 nm. Stokes’ shifts were found at 13 nm for **5** and 12 nm for **6** as expected between 10 nm and 20 and for Pc compounds.

Fluorescence measurements of standard and novel Pcs were performed at the wavelength specified for Pcs (647 nm for **5**, 615 nm for **6**). DMF for standard Pc and THF for novel Pcs were used as solvents.

The fluorescence quantum yield (Φ_F_) of unsubstituted ZnPc is known as 0.17 (41). Φ_F_ of **5** and **6** were calculated as 0.080 and 0.234 by using Eq. 1.[^23^, ^35^, ^41^]

Fluorescence lifetime (τ_F_) means the average time of the molecule at the excited state before returning to its ground state. The fluorescence (τ_F_) and radioactive (τ_0_) lifetimes are directly related to the magnitude of Φ_F_ value and they were calculated as 0.32 ns for **5** (4.13 for **6**) and 3.96 ns for **5** (17.67 for **6**), respectively. τ_F_ and τ_0_ values of **5** are lower than reference unsubstituted ZnPc (τ_F_ is 1.03 and τ_0_ is 6.05). The reason for the decrease in fluorescence life is the nature of the fluorophore and the internal transformation affected by its environment and the presence of inter‐system transition events. On the other hand, the measured values of τ_F_, τ_0_, and Φ_F_ for **6** are satisfactorily well compared to standard ZnPc. The results of the photophysical measurements of compounds **5** and **6** synthesized within the scope of this study are given in detail in Table 1.

**Table 1:** Compounds **5** and **6** synthesized in this study: Excitation and emission spectral data, photophysical parameters, and fluorescence quenching results.

Compound	Excitation λ_Ex_ (nm)	Emission λ_Em_ (nm)	Stokes’ shift ΔStokes (nm)	Φ_F_	τ_F_ (ns)	τo (ns)	k_F_ (s^1^)(×10^8^)^b^	K_sv_^c^	k_q_ (×10^10^ s^−1^)^c^
**5**	722	734	13	0.08	0.32	3.96	2.52	41.01	12.82
**6**	677	690	12	0.234	4.13	17.67	5.66	32.71	0.79
**ZnPc** ^[a]^	670	676	16.6	0.17	1.03	6.05	16.53	57.60	5.59

[a] ref. [31], [b] k_F_: fluorescent rate constant. Values were obtained with the formula k_F_=Φ_F_/τ_F_. [c] K_sv_: The Stern‐Volmer constant, k_q_: Bimolecular quenching constants.

According to the literature, there are limited studies on the photophysical measurements of phthalocyanines containing alkynyl groups. In these studies reported by Bayır et al. in 2014 and 2015, symmetric and asymmetrical phthalocyanines bearing alkynyl groups were synthesized and their photophysical properties were investigated.[^28^, ^29^] The results from these studies are summarized in Table 2 for comparison. When the fluorescence quantum yields of compounds **5** and **6** were compared with the examples in the literature, it was observed that the fluorescence quantum yield of compound **5** was close to the quantum yield of tetrasubstituted ZnPc compounds in the literature.^[28]^ In addition, the fluorescence quantum yield of compound **6** was found to be better than other compounds carrying alkyl groups in the literature[^28^, ^29^] and unsubstituted ZnPc.^[35]^

**Table 2:** Photophysical parameters, and fluorescence quenching results of Pc molecules from the literature.

Compound	Φ_F_	τ_F_ (ns)	τo (ns)	k_F_ (s^1^)(×10^8^)^a^	Reference
2, 9(10), 16(17), 23(24)‐Tetrakis‐((3,5‐difluorophenyl)ethynyl) phthalocyaninato zinc(II)	0.11	2.82	3.96	25.63	[28]
[2,9,16,23‐Tetrakis(3,5‐bis(trifluoromethyl) phenylethynyl) phthalocyaninatozinc(II)]	0.10	2.90	17.67	29.00	[28]
2,3,9,10,16,17‐Hexakis(hexylthio)‐23,24‐bis(thiophene‐3‐ylethynyl)‐29H, 31H‐phthalocyaninato zinc(II)	0.22	0.04	6.05	0.16	[29]

### Fluorescence quenching studies by benzoquinone [BQ]

**Figure 4:**
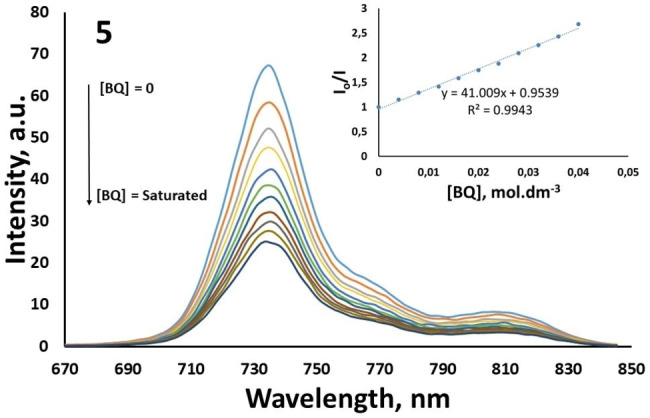
Fluorescence emission spectral changes of **5** (4.00×10^−6^ mol dm^−3^) on the addition of different concentrations of BQ in THF. [BQ]=0, 0.008, 0.016, 0.024, 0.032, 0.040 mol dm^−3^ and Stern‐Volmer plots for benzoquinone (BQ) quenching of **5** (insert figure).

**Figure 5:**
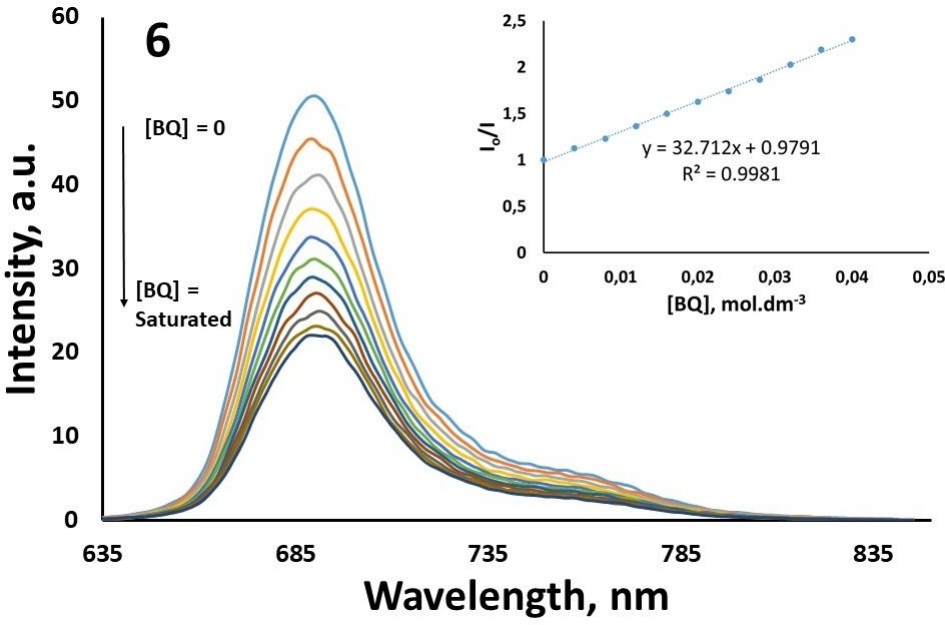
Fluorescence emission spectral changes of **6** (4.00×10^−6^ mol dm^−3^) on the addition of different concentrations of BQ in THF. [BQ]=0, 0.008, 0.016, 0.024, 0.032, 0.040 mol dm^−3^ and Stern‐Volmer plots for benzoquinone (BQ) quenching of **6** (insert figure).

The fluorescence quenching studies of **5**–**6** were performed by adding BQ to the Pcs in THF. Figure 4 and Figure 5 show the changes in fluorescence emission spectra of **5** and **6** by the addition of different concentrations of BQ, respectively. The fact that the slopes of the I_o_/I versus [BQ] graphic drawn as a result of the quenching studies of the novel ZnPcs (**5**, **6**) are in a straight line showing that the fluorescence quenching studies are in agreement with the Stern‐Volmer kinetics (insert Figure 4 and 5).

Quinones and BQ have high electron affinities and are compounds generally used in the electron transfer process.^[42]^ The energy of the excited singlet state of Pc compounds is lower than the lowest excited energy state of benzoquinone. As a result, it is not possible for energy transfer from the excited Pc compound to BQ. Hence, quenching of Pcs fluorescence by BQ occurs via excited‐state electron transfer, from MPc to BQ.[^43^, ^44^] The Ksv values were determined as 41.01 for **5** and 32.71 for **6** (Table 1). The values found for both novel Pc compounds are lower than the known value of 57.65 M^−1^ for the standard ZnPc.[^23^, ^35^] kq value was calculated 12.82×10^10^ s^−1^ for **5** and 7.92×10^9^ s^−1^ for **6** in THF by using Eq 4.

## Conclusions

In the present study, two new phthalonitrile derivatives and their corresponding peripheral octaalkynyl zinc Pcs bearing ethynylcyclohex‐1‐ene and cyclohexyl groups were synthesized and characterized for the first time. The characterization of all newly synthesized compounds was performed by using spectroscopic techniques and confirmed the proposed structures. As a result of the aggregation studies for compounds **5** and **6** in various organic solvents and at varying concentrations of THF, no aggregation was observed according to the Lambert‐Beer law. The photophysical properties of **5**, and **6** were examined and compared to each other and the unsubstituted ZnPc. The fluorescence quantum yields of **5**, and **6** were determined as 0.080 and 0.234, respectively. Also, fluorescence quenching studies were conventionally carried out in THF with the addition of benzoquinone. Stern‐Volmer kinetics were examined. Ksv values for both new compounds were found to be lower than the standard. Although the fluorescence quantum efficiency detected for compound **5** is lower than the standard, the measured fluorescence quantum efficiency and fluorescence lifetime for **6** are satisfactorily better than the standard. In this respect, compound **6** in particular has the potential to be used in many different fields, from organic semiconductors to photodynamic therapy.

## Supporting Information

Spectroscopic data of the newly synthesized compounds are provided in the supporting information file.

## Conflict of interests

The authors declare no conflict of interest.

1

## Supporting information

As a service to our authors and readers, this journal provides supporting information supplied by the authors. Such materials are peer reviewed and may be re‐organized for online delivery, but are not copy‐edited or typeset. Technical support issues arising from supporting information (other than missing files) should be addressed to the authors.

Supporting Information

## Data Availability

The data that support the findings of this study are available from the corresponding author upon reasonable request.
