# Supplementary material for: Effect of Direct Alkyne Substitution on the Photophysical Properties of Two Novel Octasubstituted Zinc Phthalocyanines
Source: ChemistryOpen. 2024 Jan 26;13(7):e202300295. doi: 10.1002/open.202300295 (PMC11230919; doi:10.1002/open.202300295)
Supplement: Supplementary file 1 — Supporting Information [file OPEN-13-e202300295-s001.pdf]

ChemistryOpen

Supporting Information

Effect of Direct Alkyne Substitution on the Photophysical Properties of Two Novel Octasubstituted Zinc Phthalocyanines

Hande Pekbelgin Karaoğlu*

Supporting Information

Figure S1. ^{1}H NMR spectrum of **3**

Figure S2. ^{13}C NMR spectrum of **3**

Figure S3. FT-IR spectrum of **3**

Figure S4. ^{1}H NMR spectrum of **4**

Figure S5. ^{13}C NMR spectrum of **4**

Figure S6. FT-IR spectrum of **4**

Figure S7. ^{1}H NMR spectrum of **5**

Figure S8. Mass spectrum of **5**

Figure S9. FT-IR spectrum of **5**

Figure S10. ^{1}H NMR spectrum of **6**

Figure S11. Mass spectrum of **6**

Figure S12. FT-IR spectrum of **6**

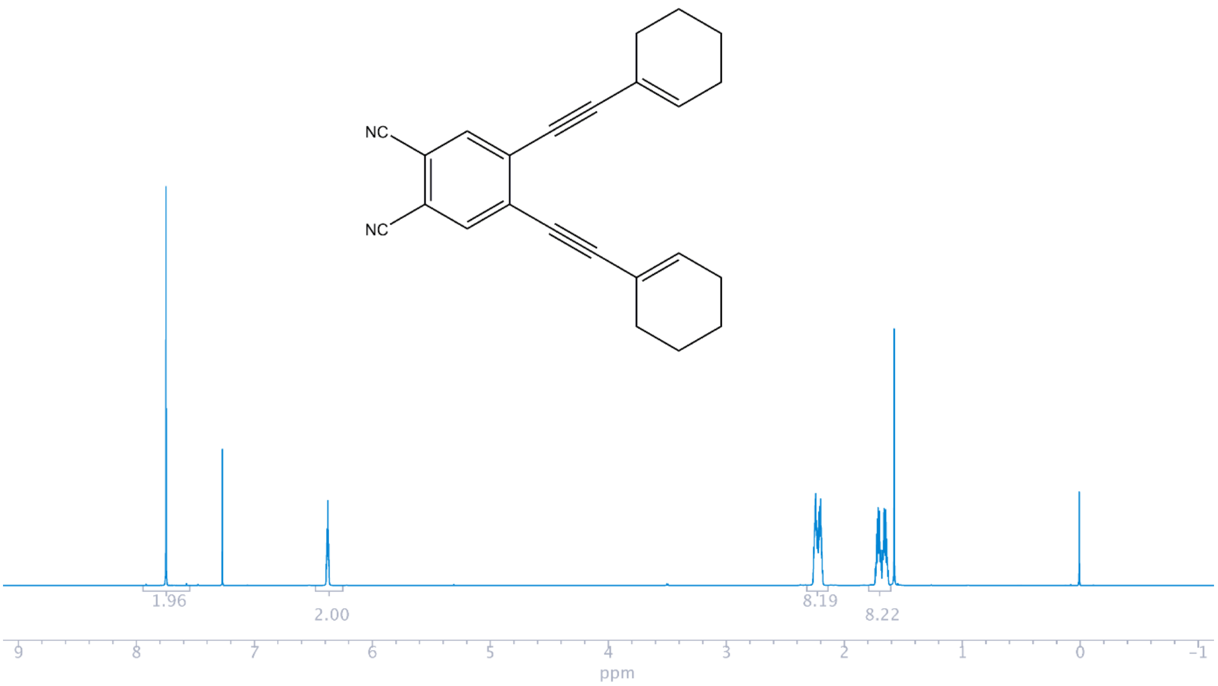

Figure S1. ^{1}H NMR spectrum of **3**

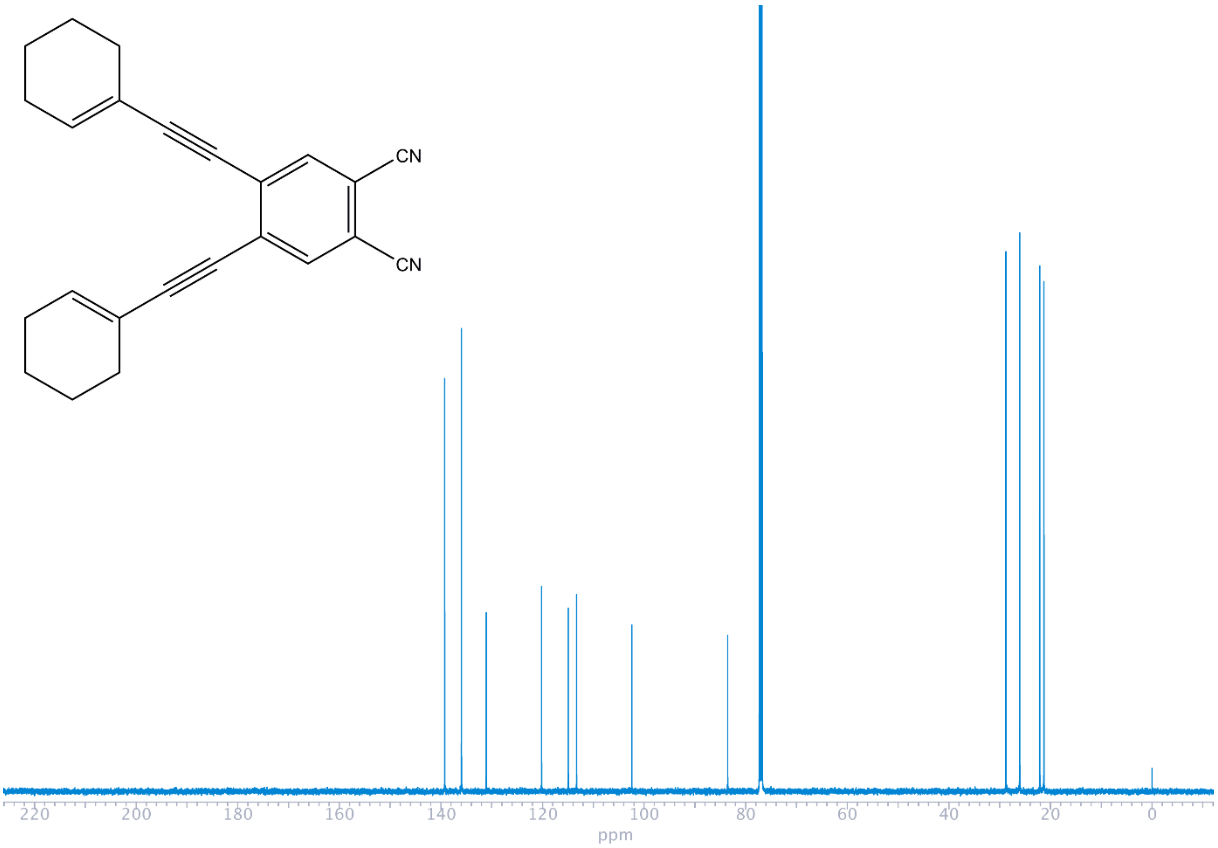

Figure S2. ^{13}C NMR spectrum of **3**

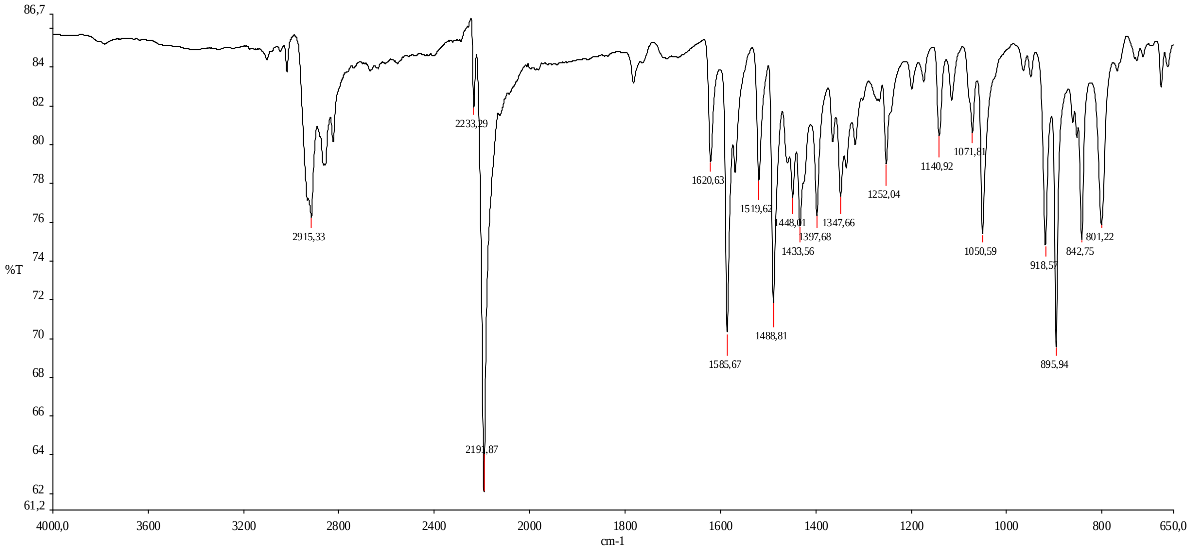

Figure S3. FT-IR spectrum of **3**

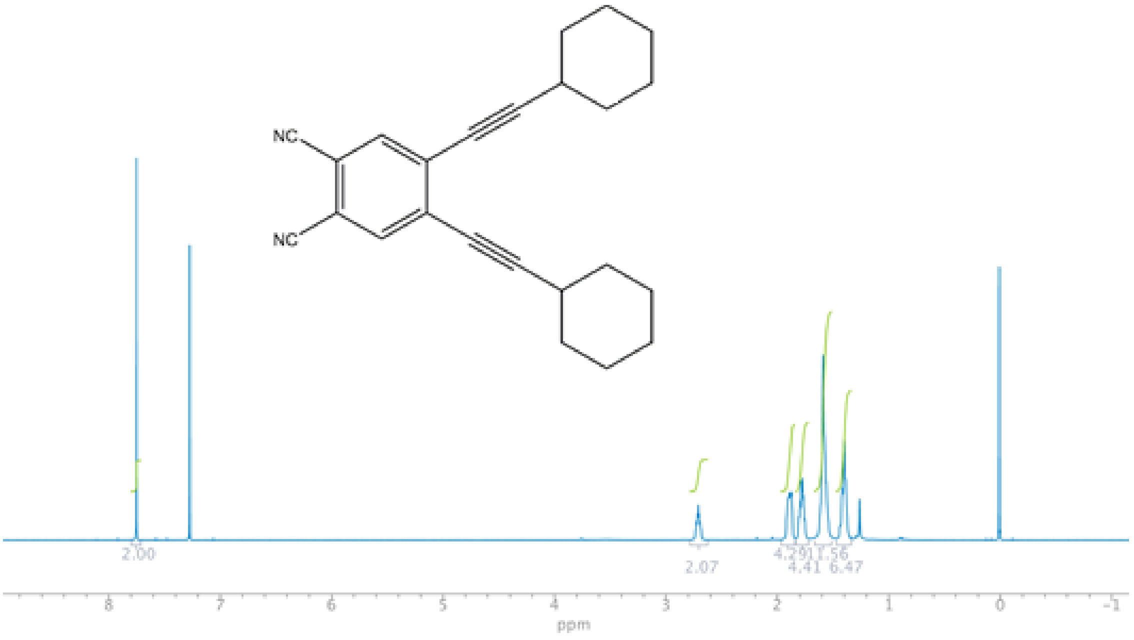

Figure S4. ^{1}H NMR spectrum of **4**

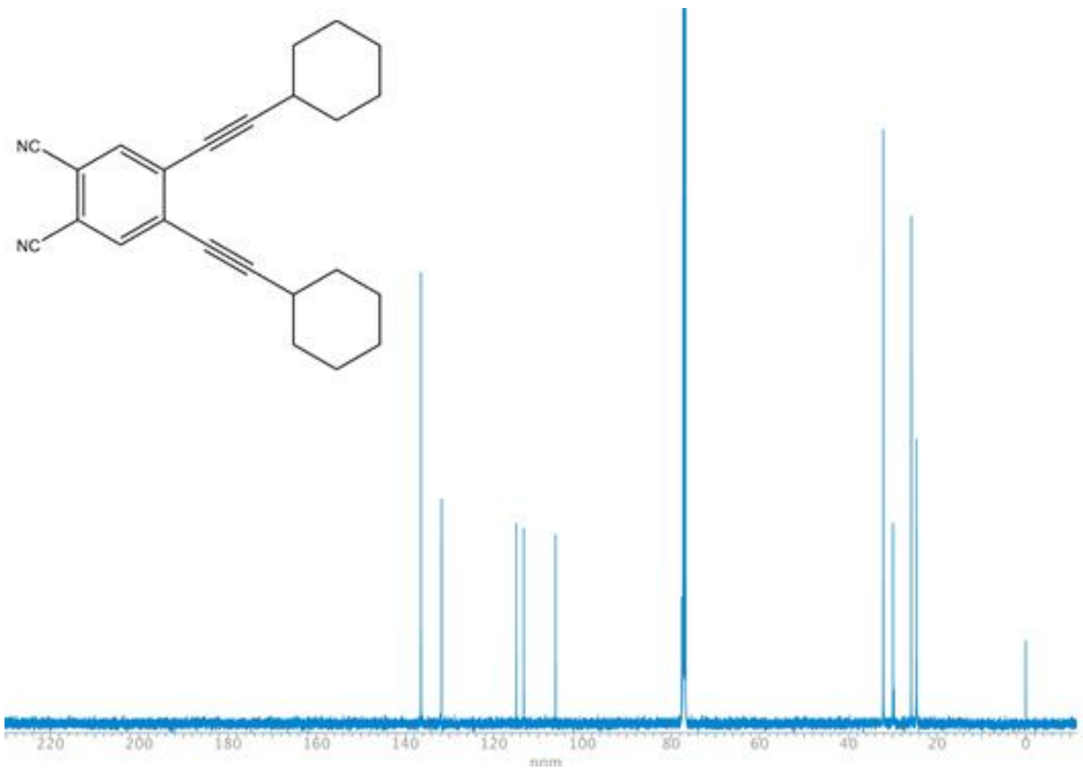

Figure S5. ^{13}C NMR spectrum of **4**

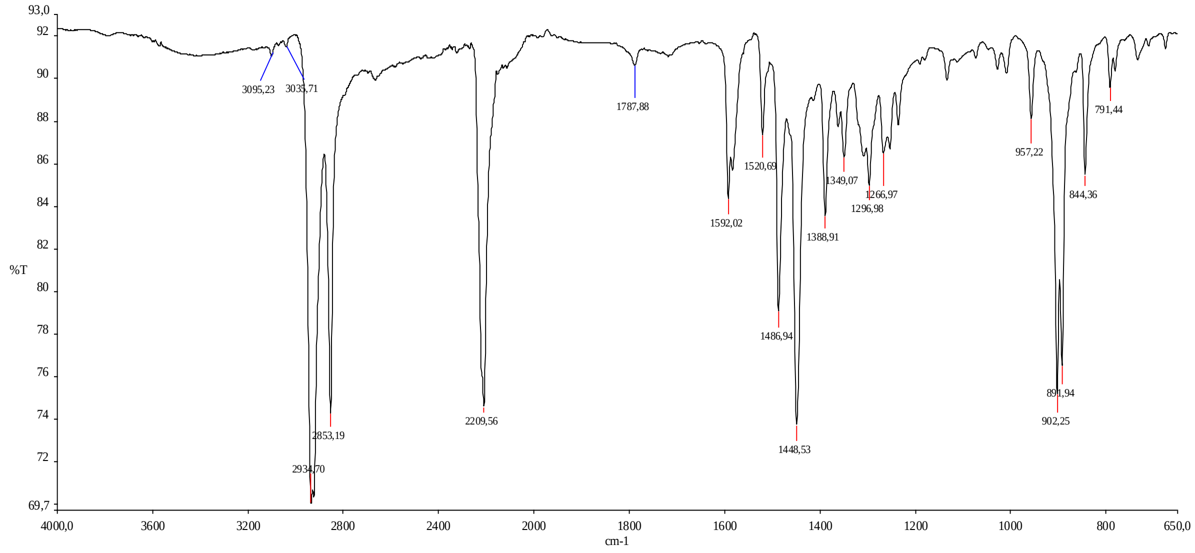

Figure S6. FT-IR spectrum of **4**

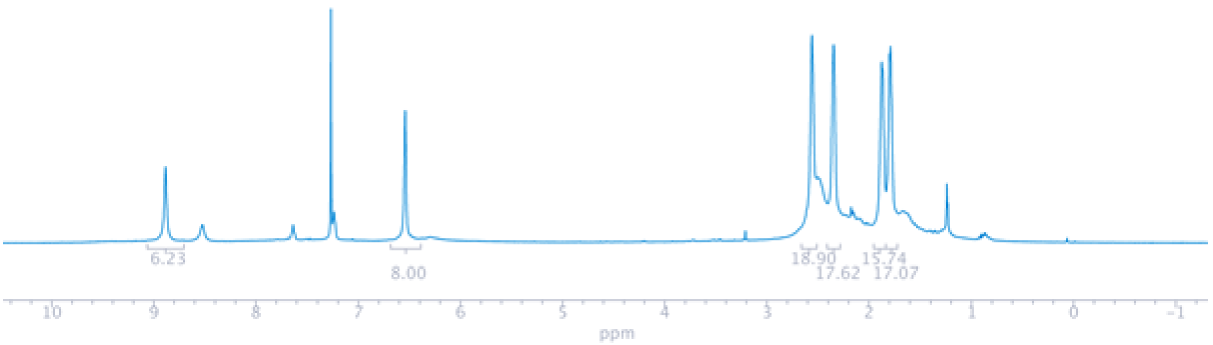

Figure S7. ^{1}H NMR spectrum of **5**

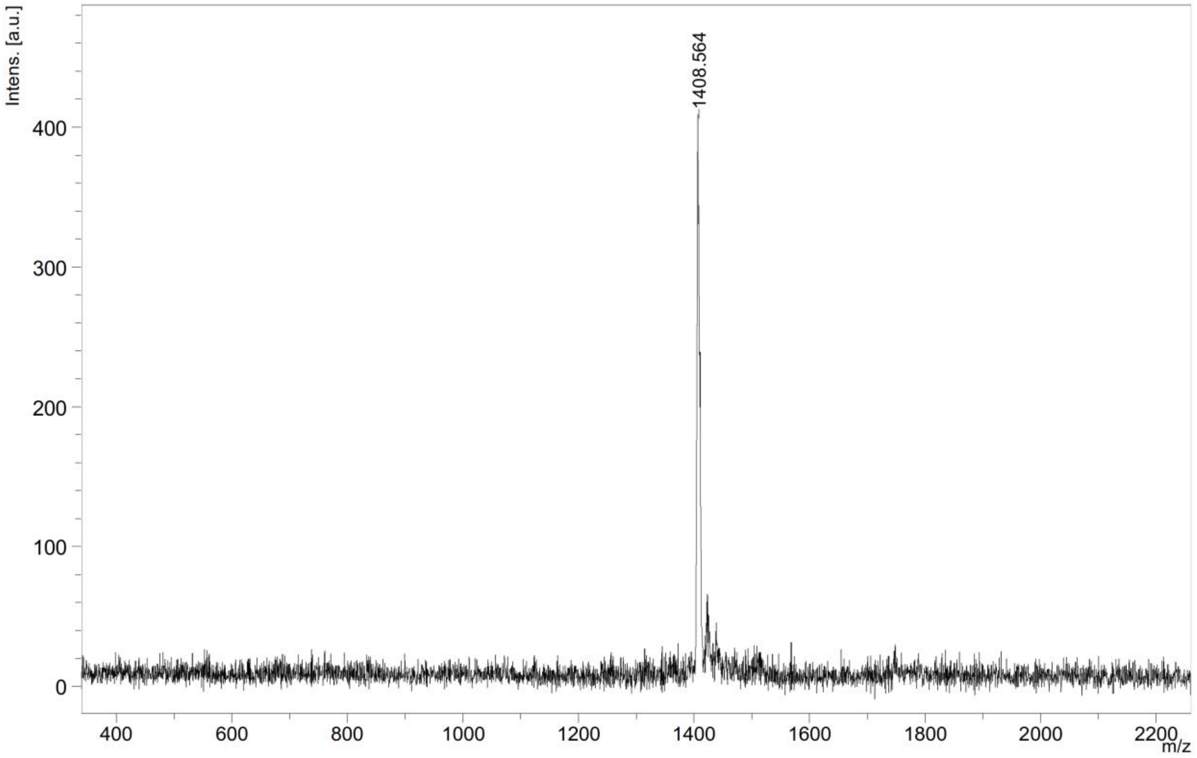

Figure S8. Mass spectrum of **5**

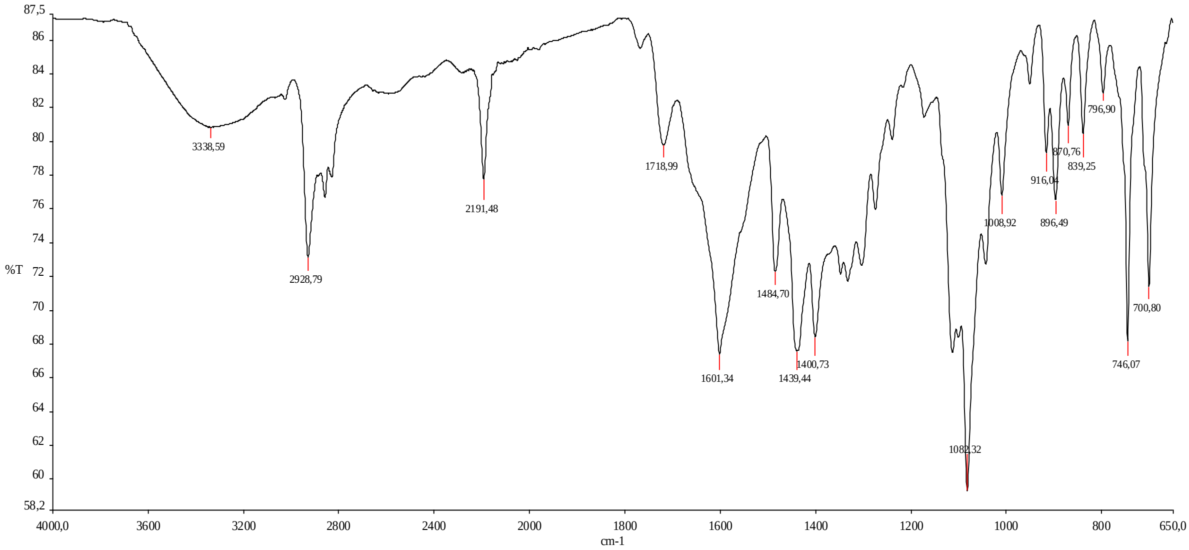

Figure S9. FT-IR spectrum of **5**

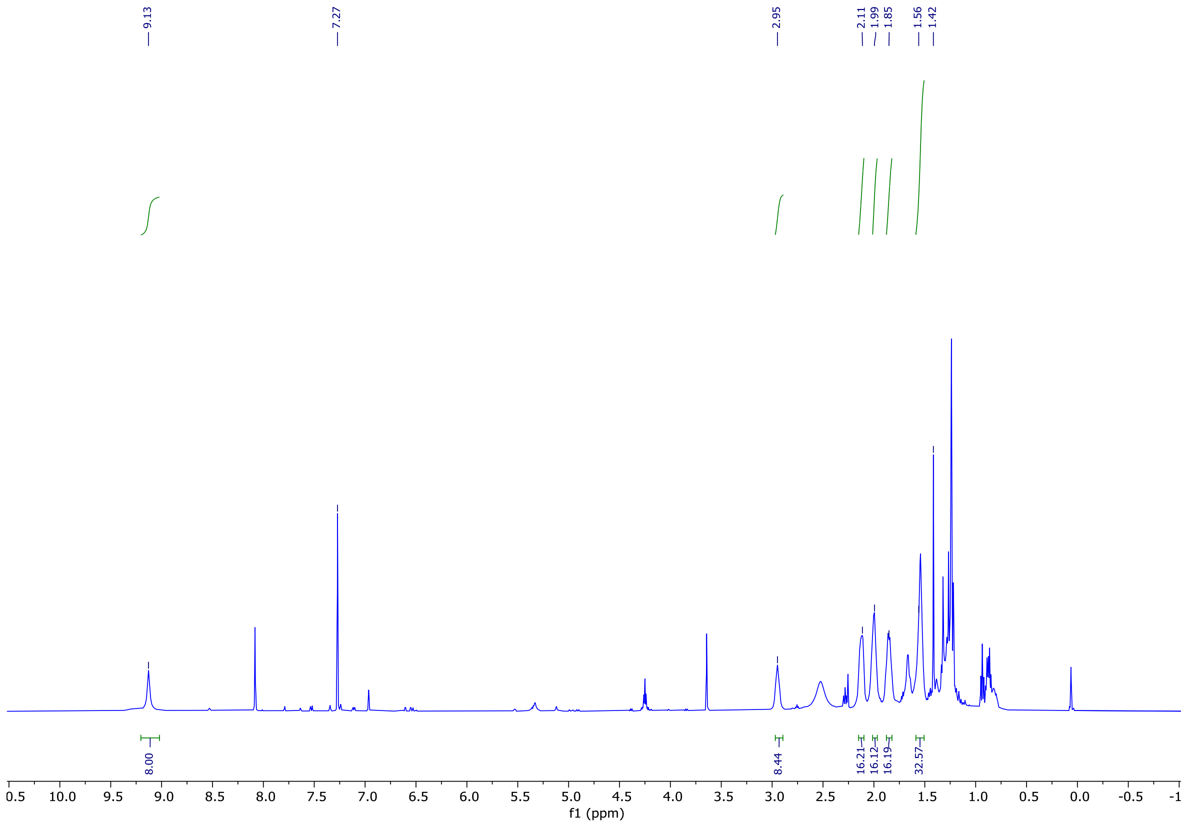

Figure S10. ^{1}H NMR spectrum of **6**

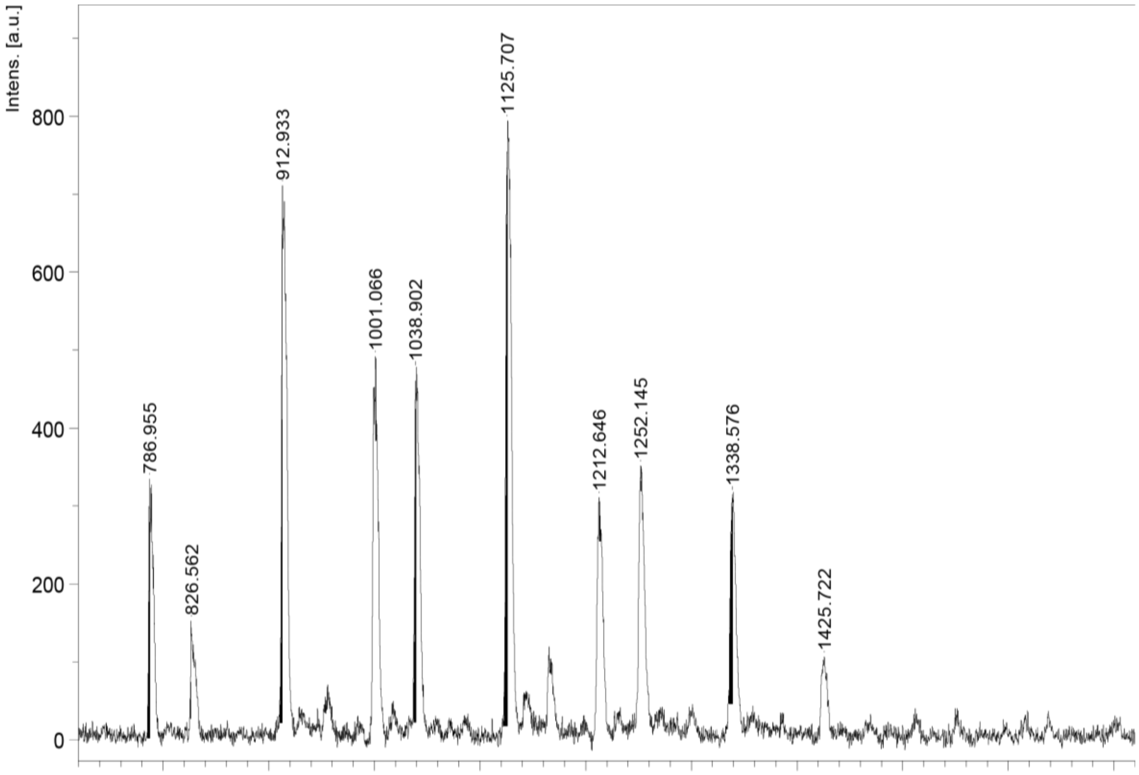

Figure S11. Mass spectrum of **6**

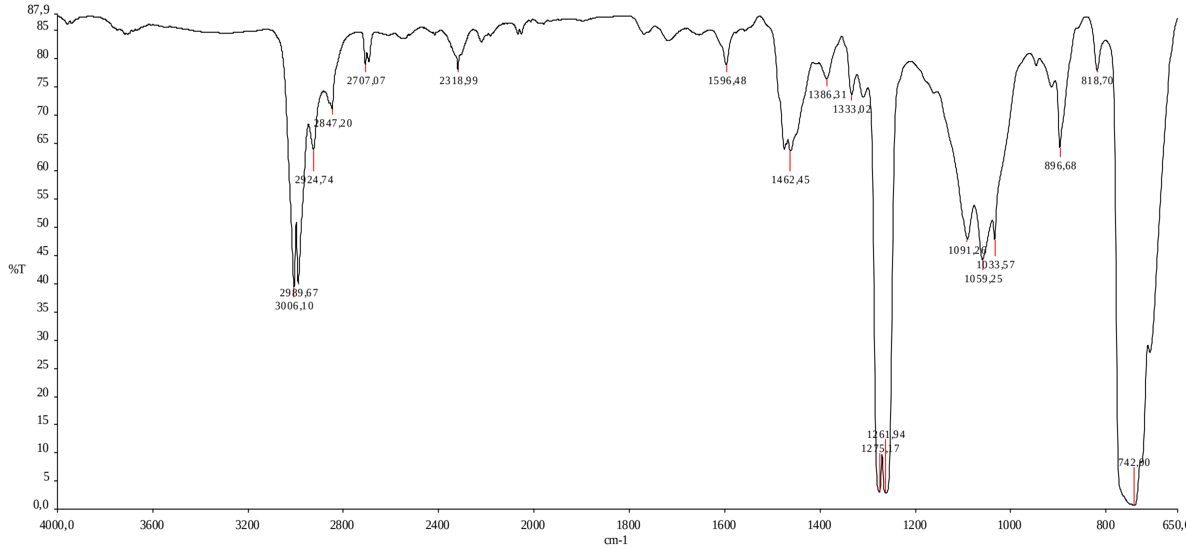

Figure S12. FT-IR spectrum of **6**
